# A new approach for reconstruction of the gunshot defect of the flexor surface of the ungual (distal) phalanx by the proper transverse branch of the digital artery: a case report of combat patient injured in the Russo-Ukrainian war

**DOI:** 10.1186/s13049-023-01139-0

**Published:** 2023-10-30

**Authors:** Serhii V. Tertyshnyi, Igor Lurin, Igor P. Khomenko, Kostiantyn V. Gumeniuk, Volodymyr Yu. Shapovalov, Volodymyr V. Nehoduiko, Maksym Gorobeiko, Andrii Dinets

**Affiliations:** 1 Military Medical Clinical Center of the South Region of the Ministry of Defense of Ukraine, Odesa, Ukraine; 2 National Academy of Medical Sciences of Ukraine, Kyiv, Ukraine; 3 State Institution of Science “Research and Practical Center of Preventive and Clinical Medicine”, State Administrative Department, Kyiv, Ukraine; 4 National Military Medical Clinical Centre of the Ministry of Defense of Ukraine, Kyiv, Ukraine; 5 Department of Surgery #4, Kharkiv National Medical University, Kharkiv, Ukraine; 6 Department of Healthcare, Kyiv Agrarian University, Lancet XXI, Arsenalna str., 9/11, Kyiv, 01011 Ukraine

**Keywords:** Perforating flaps, Dynamic digital thermography, Distal transverse digital artery, Hand injury

## Abstract

**Background:**

Gunshot injury to the hand is severe trauma, requiring complicated reconstruction surgery for the damaged anatomic site to restore all the hand functions. The aim of this study was to show the example of the distal phalanx reconstruction by using a flap with distal transverse digital artery (DTDA) blood supply as well as to demonstrate the utility of the audio Doppler application at the reconstruction stage in the combat patient injured in the Russo-Ukrainian war.

**Case presentation:**

In this report, we present a case of a 26-year-old service member of the Ukrainian Armed Forces delivered to the Military Medical Clinical Centre on the fourth day after the gunshot gutter shrapnel wound of the distal flexor of the 2nd digit with a gunshot fracture of the ungula (distal) and middle phalanges of the 2nd digit of the right hand along with a bone deficiency of the osseous structure of the distal and middle phalanges, volar soft tissues. The dorsal metacarpal artery (DMCA) flap is a universal variant among the tools of the reconstructive plastic surgeon engaged in reconstructing defects of the digital dorsum and flexors with a limited range of flaps. We consider this to be a key that conforms with the majority of the reconstructive principles, such as ‘analog replacement’, and which is simple, adequate, and easy for operating with a minimal sequela of the donor site.

**Conclusions:**

The distal transverse digital artery (DTDA) could be considered for hand reconstructive surgery for repairing defects of the flexor surface of the digit injury and hands after severe gunshot injury.

## Background

Various kinds of gunshot injuries are common in armed conflicts and warfare, causing severe trauma both to the military personnel and the civilian population [1]. A war of Russia against Ukraine was initiated in 2014 as a hybrid warfare, and an active invasion phase has been ongoing since February 24, 2022. Since then, different types of injuries have been described, and these injuries were associated with application of the high-energy weapons such as multiple-launch rocket systems, cruise missiles, or autonomous pusher-prop drones by the aggressor [2–5]. Severe injuries could be seen with various presentations, with frequent atypical or unusual clinical courses, which might be related to the bullet type as shown in experimental studies [6, 7]. Severe trauma cases were described for the genitourinary, abdomen, vascular structures, chest, and neck as well as for the extremities in war in Ukraine [2–5, 8]. It is worth to mention that gunshot injury to the upper extremity is also frequent in armed conflicts, and such kind of injury is associated with poor prognosis for post-injury recovery of hand functions [9]. The management of gunshot wounds to the upper extremity is well-described in patients injured in previous wars, but little is known about the clinical course of such trauma in the ongoing war in Ukraine. It is also worth emphasizing the fact that all kinds of hospitals (both combat and civilian) at all Levels of medical care are at high risk of attacks by cruise missiles or drones from the Russian army, which is a violation of humanitarian international law [3]. Such circumstances make it difficult to provide medical care for patients with gunshot trauma, including patients requiring a microsurgical approach for gunshot trauma to the hand.

In our everyday war surgery practice, we consider a few variants for reconstructing the digital dorsal defects, and our approach is in contrast to the known variants available for volar defects of distal parts of digits. These kinds of available variants of digital dorsal defect reconstructions of hand injury might be different: dorsal transposition or rotation flaps, distal sliding, or rotary flaps, which depend on the trauma features. Also, one should consider a two-stage reconstruction technique (e.g. reverse transverse digital flap), which is a complex approach due to transpositioning an inconsistent palmar ‘bare’ skin (e.g. island neurovascular flaps) [10].

The suggested microsurgical approach is about the free flap transposition (i.e. free flap technique), which is an attractive, but complex surgical approach. The available published data has described the dorsum of the hand to be a recipient of flaps, but not the donor’s site. In such an approach, the dorsal metacarpal artery (DMCA) flap is considered a universal variant among the others for reconstructive and plastic surgery to be engaged in reconstructing defects of the digital dorsum and flexors with a limited range of flaps. From the historical point of view, it is worth mentioning that the first presentation of the perforating flap of the DMCA as a distal perforating flap was made in 1990 [11]. The presented case by Maruyama et al. in 1990 was a variant of DMCA flaps to be taken as a flap with reflux or a distal DMCA flap (reverse dorsal metacarpal artery flap) and the DMCA is tied proximally and included into the flap with two assistant veins [12]. However, hand reconstruction using a flap supplied by a distal transverse digital artery (DTDA) might be a good alternative to known techniques [13]. We also consider such an approach to be useful in digit injury, including digit tip injuries in combat patients. Also, little is known about the digit reconstruction in the ongoing Russo-Ukrainian war.

The aim of this study was to show the example of the distal phalanx reconstruction by using a flap with distal transverse digital artery (DTDA) blood supply as well as to demonstrate the utility of the audio Doppler application at the reconstruction stage in the combat patient injured in the Russo-Ukrainian war.

## Case presentation

The clinical case is the presentation of an injured service member of the Armed Forces of Ukraine who was wounded by artillery shelling in East Ukraine in June 2022. As a result of the injury, a defect of the volar surface of the 2nd right-hand digit at the level of the distal phalanx sized 2.0 × 1.5 × 0.3 cm in zone I was diagnosed [14]. Active movements in the distal interphalangeal joint were absent due to the Kirschner wires fixation and limited in the proximal interphalangeal joint due to the pain. The sensation and microcirculation in the distal phalanx were reduced. The soft tissue tone and turgor were increased due to the circular infiltration (caused by the injury and inflammation). The following examinations were performed: clinical chemistry, blood group test, Rh factor test, blood clotting tests, X-ray of the hand in two projections, DDT of the injured area and surrounding soft tissues, and bacterial swab test of the wound surface. After a temperature scan of the wound surface, the transverse branches of the digit artery of the 2nd digit on the dorsal surface were identified. Given the size of the defect of the flexor and the anatomic structural specifics of the digit, we proposed a ‘closing’ technique using the propeller flap with a 180-degree rotation on the transverse branch of the digital artery at the level of the distal interphalangeal joint. The defect of the digital flexor resembled an overturned plate with its bottom upwards. The wound bed was presented with bone fragments of the distal and middle phalanges (yellow-white color) and the surrounding soft tissues as gray-pink granulations with fragments of partial necrosis in the area of the nail plate. The fractured fragments of the ungual and medial phalanges were fixed with two Kirschner wires.

**Fig. 1:**
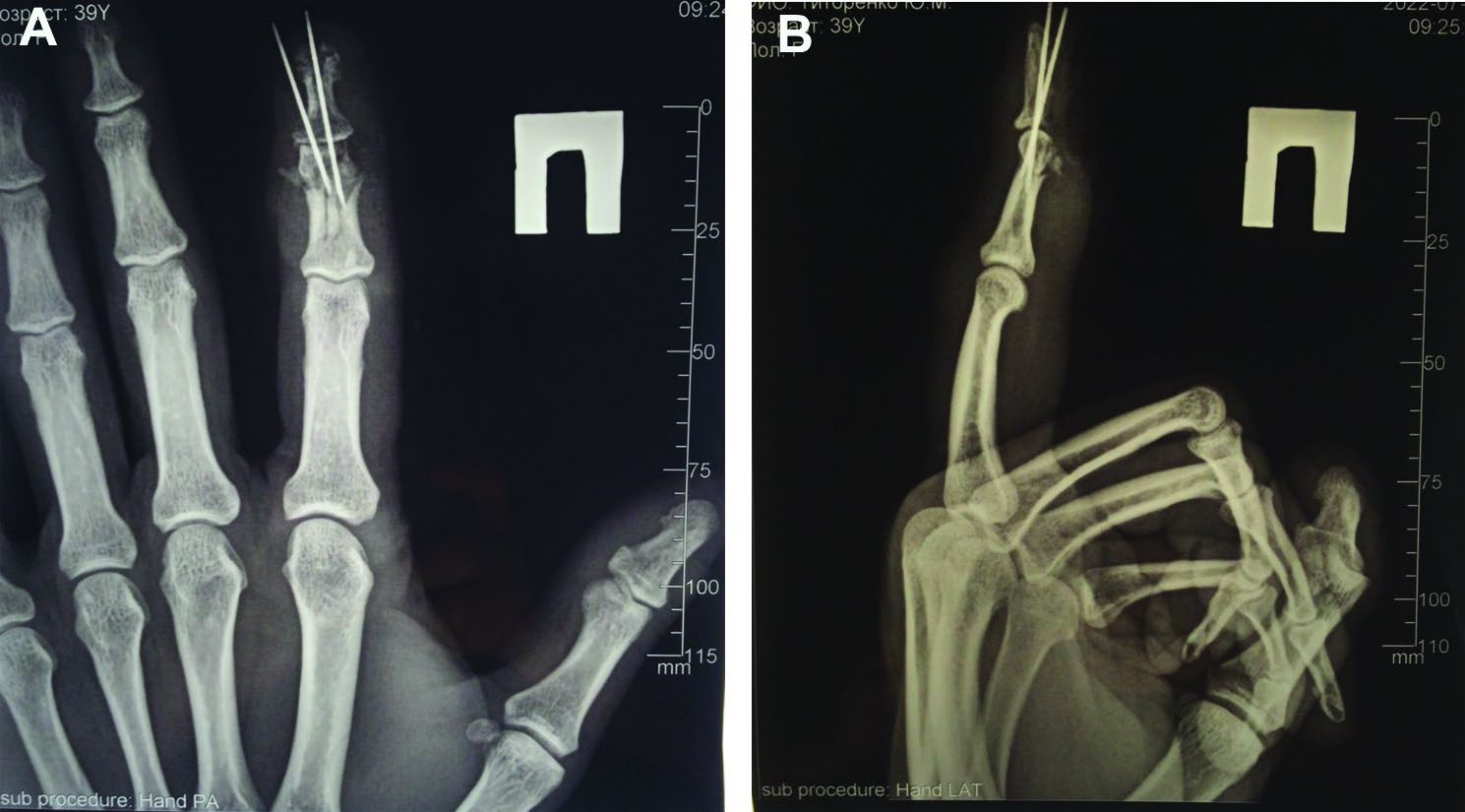
Illustration of X-ray films of the right hand in anteroposterior position (**A**), and lateral position (**B**) of the injured digit

**Fig. 2:**
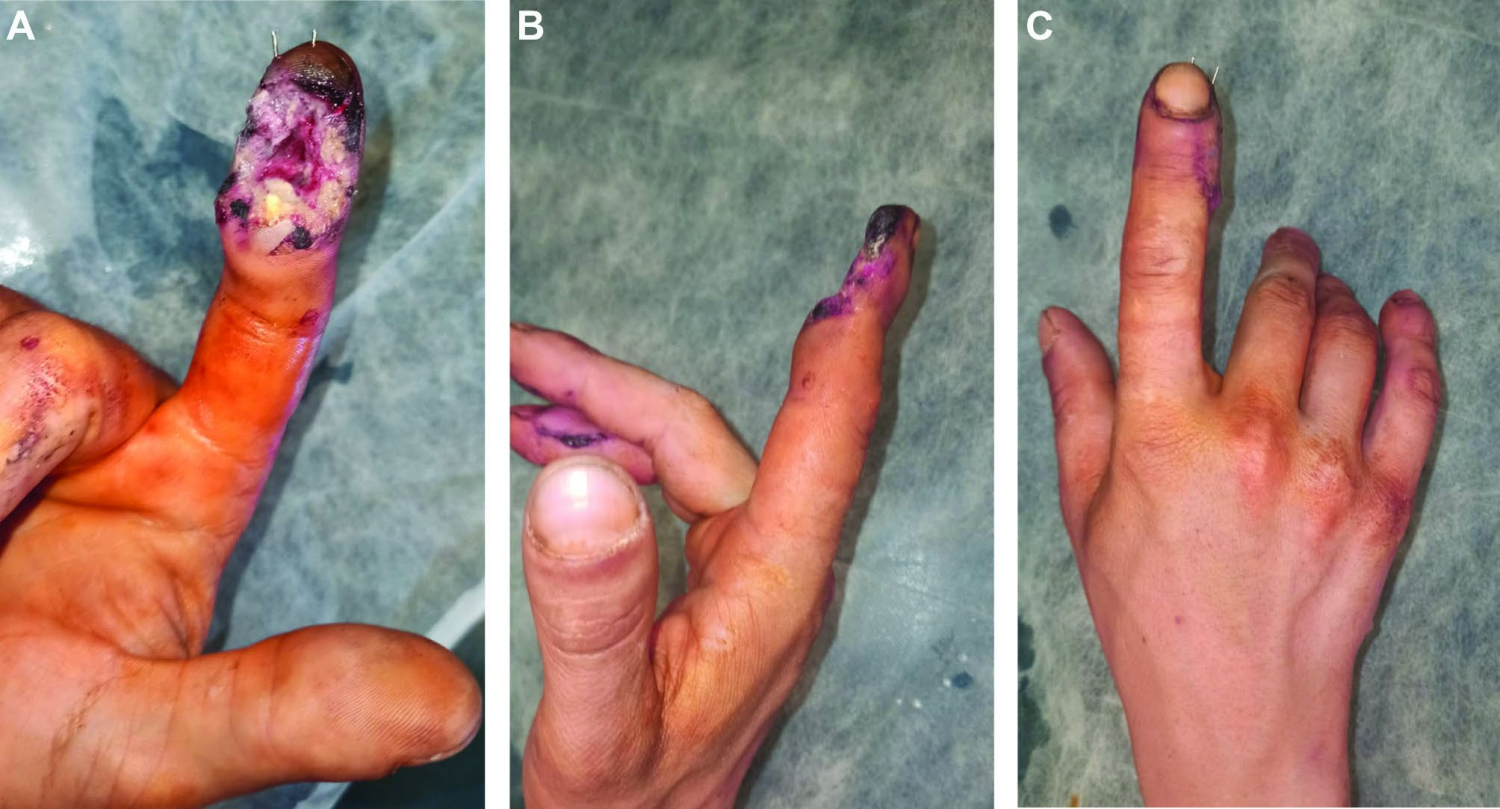
Photographs showing the defect of the volar soft tissues of the 2nd digit of the right hand (**A** – flexor, **B** – lateral view, **C** – extensor of the 2nd digit of the right hand) on the 5th day after the injury

**Fig. 3:**
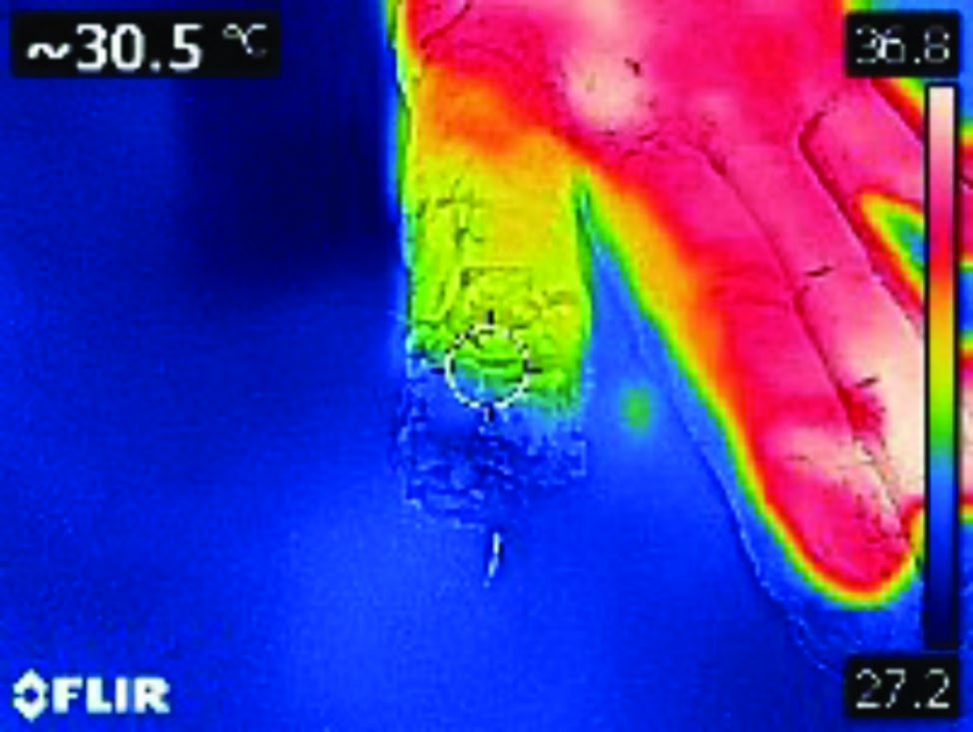
Dynamic digital thermography of the wound surface of the righthand flexor surface showed sufficient perfusion

**Fig. 4:**
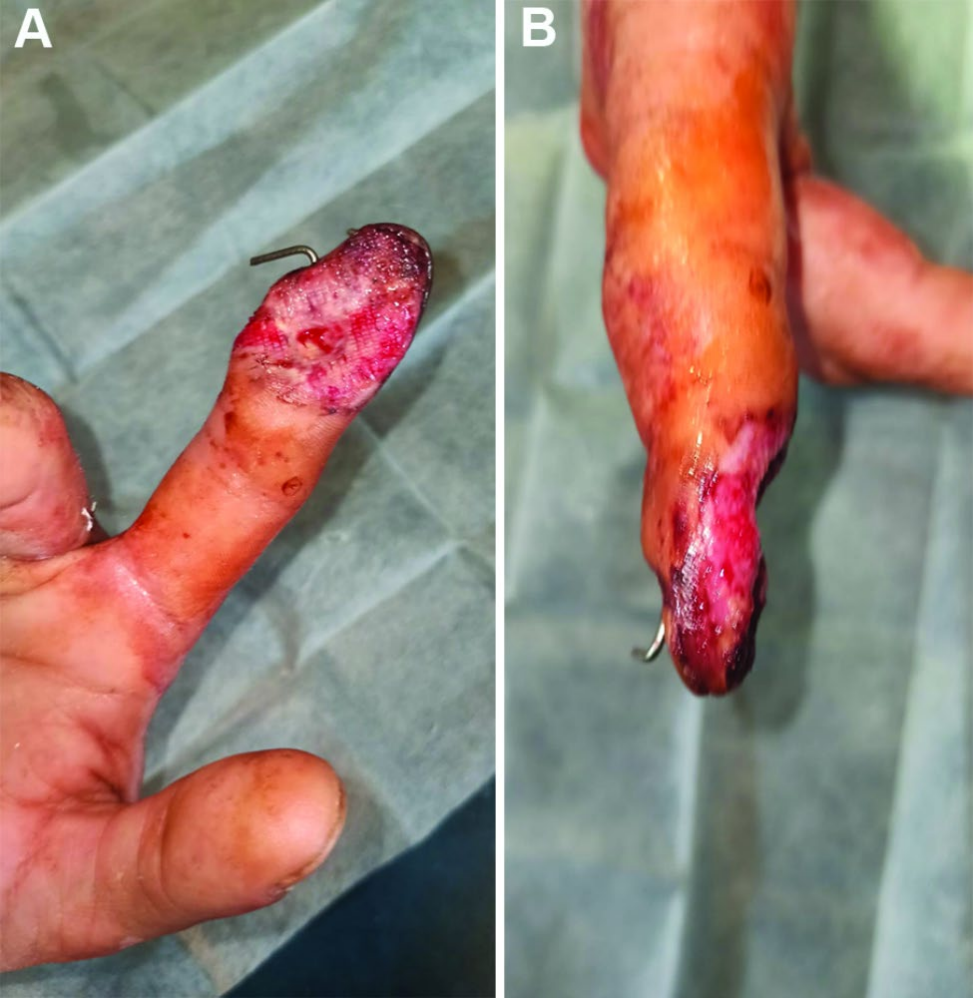
Photographs of the wound on the 6th day after the operation (**A** – flexor, **B** – lateral surface of the 2nd digit of the right hand)

The wound was sustained as a result of the adverse artillery shelling in East Ukraine (Donbas area) in July 2022. At the second level of medical care, the hand wounds were treated with CPR and Kirschner wires fixation (Fig. 1). In 4 days from the time of injury, the patient was admitted to the Southern Regional Military Medical Clinical Centre (Odesa), where the final reconstruction of the gunshot defect was performed (Fig. 2). Preoperatively, the injured hand underwent limb salvage, which was represented by the following actions: wound debridement with a maximum effort to preserve a tissue, which was performed with a mean temperature of 30.50 °C on the wound surface (Fig. 3). Sufficient blood supply was confirmed by employing an audio Doppler. Then, the pulsed lavage was used with a 0.9% sodium chloride saline in a volume of 1.0 L, which made it possible to remove tiny fragments of necrotic tissues and reduce the quantitative bacterial load of the wound surface. The final “preparatory” component was the application of bandages using hyaluronic acid, which was performed on the 6th day after the injury (Fig. 4). This surgical approach made it possible not only to preserve a larger volume of the wound defect but also to attain an active granular tissue growth to reduce the wound surface by 10% as compared with its size in admission.

**Fig. 5:**
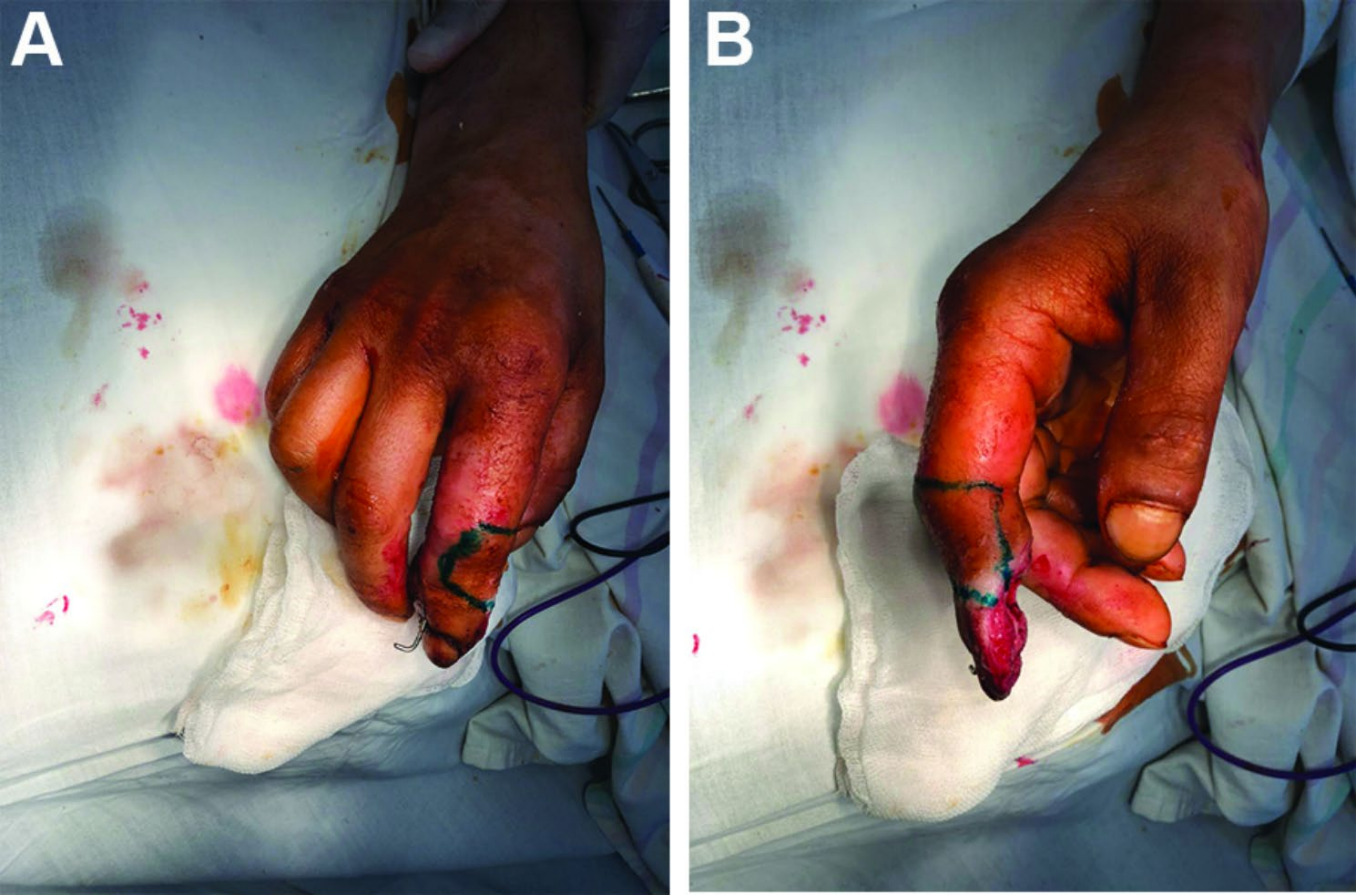
Photograph of the preoperative marking of the flap donor site (**A** – dorsal view outside the flap, **B** – lateral flap view)

The reconstructing-restoring approach was based on a multimodal approach involving distance digital thermography (DDT), which enabled to remote identification of the location of the perforating branch of the distal transverse digital artery (DTDA) at the level of the medial phalanx of the 2nd digit of the right hand. The audio Doppler was performed at the preparatory stage to confirm the location of the DTDA, and the expert-quality Doppler ultrasound provided an opportunity to assess the volumetric and velocity blood flow given the secondary changes after wounding and to additionally determine anatomical characteristics to choose the flap size (Fig. 5).

The flap area was considered only with the need in the donor zone of the flexor surface of the ungual phalanx of the 2nd digit of the right hand.

The operation was performed under the general anesthesia. A surgical binocular loupe was used with magnification x6. To prevent the active bleeding from the donor site, a pneumatic tourniquet was placed on the right upper extremity at the level of the middle third, which significantly helped to visualize the perforator during the ‘lifting’ of the flap and to perform a delicate dissection.

Before the perforator was exposed, which was marked on zone I of the hand (at the level of the distal interphalangeal joint), a pneumatic tourniquet relaxation and blood flow assessment by using the manual audio Doppler were performed. This helped to monitor the angioarchitecture of the flap feeding.

In the second stage, the flap transposition to the DTDA was performed according to the classic technique, from the proximal part of the flap. The superficial veins that ran along the axis of the flap after the ligatures applied in the proximal section were incorporated into the flap. Preserving the paratenon of the extensor tendon of the 2nd digit was a prerequisite for the reconstruction.

In the distal section of the flap, the small veins around the flap stalk were preserved to avoid venous stasis in the postoperative period. After lifting the flap proximally and distally, the pneumatic tourniquet was completely disconnected.

**Fig. 6:**
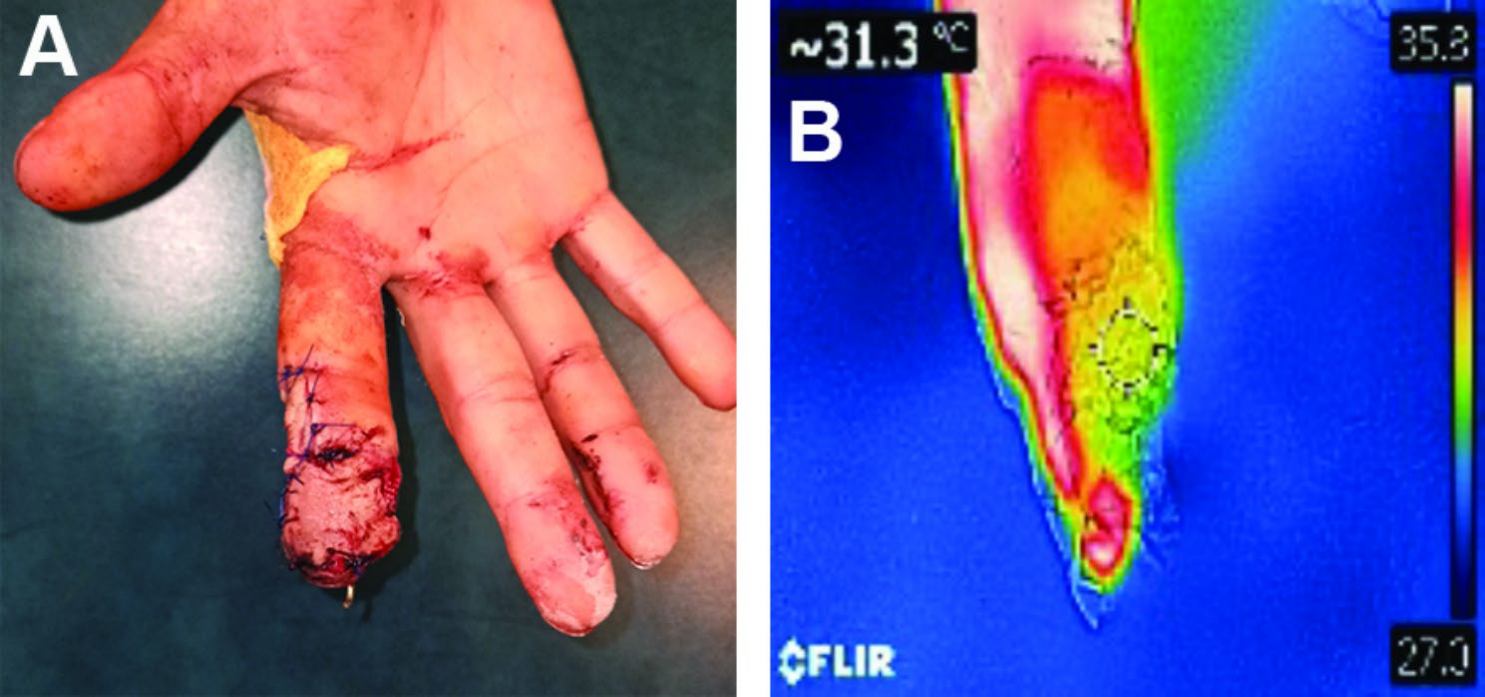
Postoperative photograph of the injured digit after the flap transfer: **A**. Flap with 180º positioning, **B** – thermographic monitoring showing sufficient perfusion

When the flap was lifted, the temperature on the surface of the flap was checked using the DDT, and the presence of blood flow was checked with the audio Doppler used for 20 min. The flap was then rotated at 180 degrees and fixed to the wound defect with simple interrupted suture placement (Fig. 6). The tension-free fixation of the flap was mandatory to avoid damage to the vascular base of the feeding. After the flap fixation, the multimodal monitoring of the reverse blood flow quality was performed: DDT + audio Doppler for 10 min. The donor site was closed with a split skin flap taken from 1 interdigital space. A plaster splint was placed on the extensor surface of the right hand in the physiological position of flexion.

**Fig. 7:**
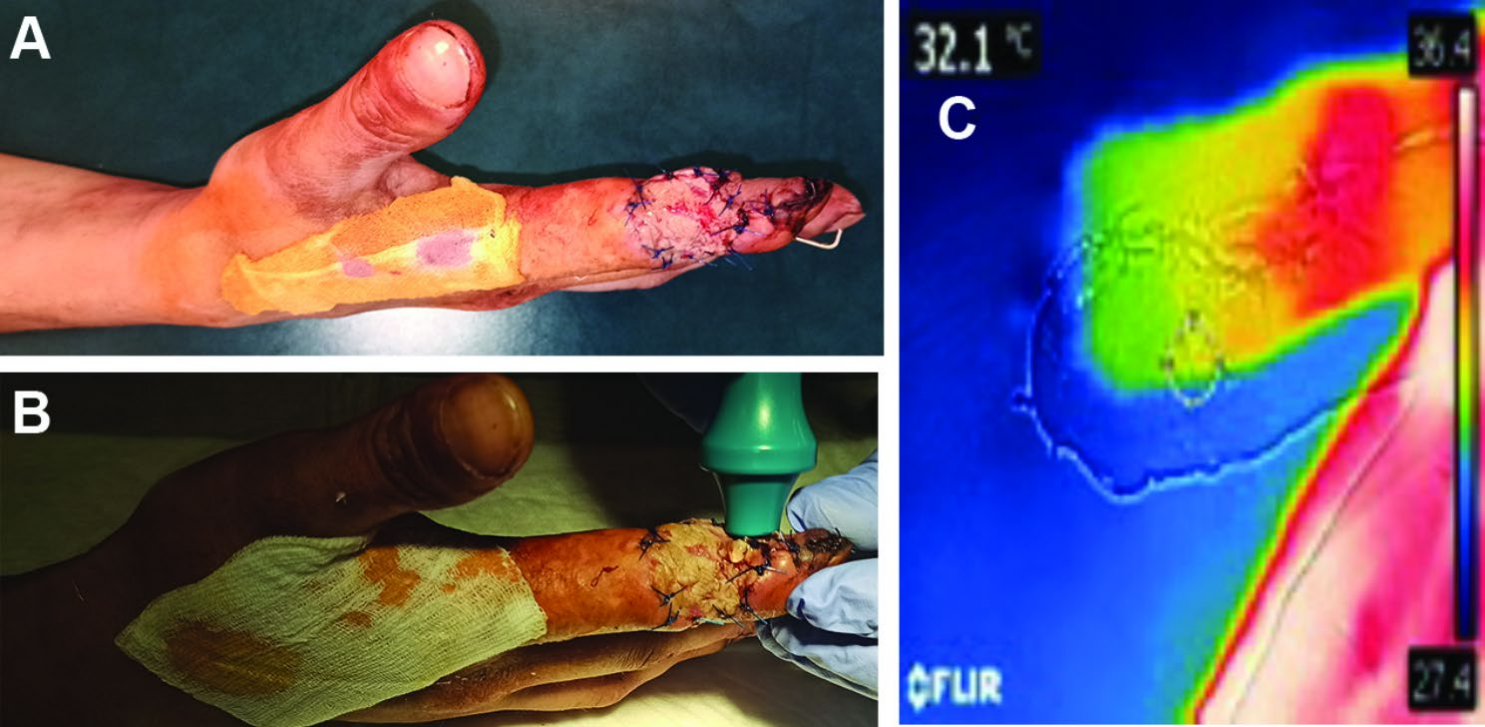
Illustration of the injured digit on the 1st day after the reconstruction using the propelling flap on the DTDA, rotation of the reverse flap at 180 degrees. **A** – lateral view, **B** – dynamic digital thermography, **C** – thermographic monitoring showing sufficient perfusion within the reconstruction area

The postoperative monitoring of the flap for 1 day was performed every 6 h according to the multimodal pattern (DDT + audio Doppler), which confirmed the pulsation in the rotated vascular structure (Fig. 7).

The patient underwent the following anticoagulation therapy: enoxaparin 4000 anti-xa iu/0.4ml per day during 10 days, followed by rivaroxaban 10 mg**/**day during 21 days.

Referring to the technique of reconstruction of a gunshot defect of the 2nd digit of the right hand, the defect was closed in 3 weeks. The donor site was closed using the skin graft technique. After the wound healing by primary tension, the medical leave was prescribed to the patient for 30 days.

## Discussion

Reconstructing gunshot defects of zone I of the hand remains difficult for reconstructive plastic surgeons, as it is important for a transplanted tissue not to be soft as the flexor skin must resist shear and pressure forces. The war conditions are also considered an obstacle to provide an adequate microsurgical intervention within the appropriate time after the injury because of interruptions in the medical evacuation by Russian strikes on medical convoys and other medical facilities in the ongoing Russo-Ukrainian war [3–5, 8, 15–17].

There are several other variants to perform the hand reconstructions. For example, Quaba flap is based on the perforator vessels which is taken at the level of the neck of the carpal bone by approximately 0.5-1 cm more proximally to the metacarpal-phalangeal joint and distally from the juncturae tendinum. Another variant described in the literature refers to the dilated flap based on the distal junction of the dorsal carpal system and dorsal branches of the digital artery at the level of the medial proximal phalanx [10–12].

The options for reconstructing defects of the flexor surface of the digit are limited, and some of the variants include rotation, advancement, and transposition of flaps even for small defects. Defects of moderate size can be “closed” with reversible DTDA flaps and adipofascial flaps for rotation [18–22]. When large defects are detected it is also possible to use the abdominal flap or superficial circumflex iliac artery perforator flap [23, 24].

The research also demonstrated that using the DDT with the audio Doppler is a suitable and quick method for identifying and assessing the properties of the perforating system of the hand. In reconstructing defects of the palmar surface of the hand and/or digits, we consider certain principles. The palmar and dorsal skin is functionally and anatomically different; the dorsal skin is subtle, flexible, and movable, which provides for a free and sliding movement of the lower tendons and joints of the extensors. The aesthetic parameters should also be considered in planning flaps in these areas according to the following: color compatibility, texture compatibility, hairiness, final cicatrix location and development of skin contractures, and cosmetic properties of the donor site [19].

Our case is a demonstration that flaps from the hand dorsum are adequate and suitable for reconstruction surgery. More than one flap is possible to transposite for closing several defects of the dorsum and flexor of the digits. The lower flap is located predictably, longitudinally along the intermetacarpal spaces with a regular anastomosis with the palmar carpal and proper digital arteries, as opposed to the palmar blood flow with several vessels, but the location is chaotic, and anastomoses are minimal. The flap can be taken as a vascularized bone or tendon flap for reconstructing osseous defects. Adhesion of the donor site is minimal since it can be closed primarily in the form of a linear suture in most cases [10, 11, 19]. We do not consider any time limits to perform the DTDA flaps reconstruction, however one should consider possible thrombosis of the magistral vessels of the upper extremity or significant scarring of the wound area, which are limitations. In our opinion, this reconstruction method can be applied for all kinds of gunshot injury, because of blood supply from more proximal arteries is more capable as compared to blood supply form distal arteries. However, the possible limits for application of this method might be considered for severe burns.

The DTDA flaps reconstruction is highly efficient, however it might be associated with such early complications as oosteomyelitis of the phalanx, wound infection, thrombosis of the digital arteries in case of rotation over 180° (i.e. overrotation) or flap twisting (both resulting in narrowing of arteries lumen). The possible late complication might be posttraumatic deformative arthritis of distal interphalangeal joint in case of skipping physiotherapy exercises during the rehabilitation process.

## Conclusions

The distal transverse digital artery (DTDA) could be considered for hand reconstructive surgery for repairing defects of the flexor surface of the digits and hands after severe gunshot injury.

Our clinical case is based on the experience obtained in providing surgical assistance in the Armed Forces of Ukraine to the injured at the stage of a surgeon`s medical evacuation, which introduces bias into the study design. Employing a multimodal pattern for monitoring gunshot defects of the upper extremity has demonstrated its effectiveness and qualitative addition to the existing methods of observation in the course of reconstructive and restorative operations.

## Data Availability

All data regarding this case report has been reported in the manuscript. Please contact the corresponding author in case of requiring any further information.
